# Personalized Computational Fluid Dynamics Analysis of Cerebral Venous Hemodynamics in a Case of Deep Cerebral Vein Thrombosis

**DOI:** 10.3390/jpm15120570

**Published:** 2025-11-28

**Authors:** Adisu Mengesha Assefa, Lina Palaiodimou, George Bourantas, Antonis Sakellarios

**Affiliations:** 1 Department of Mechanical Engineering and Aeronautics, University of Patras, 26504 Patras, Greece; adisu.mengesha@upatras.gr; 2 Second Department of Neurology, National and Kapodistrian University of Athens, 11527 Athens, Greece; lpalaiodim@med.uoa.gr; 3 Department of Agriculture, University of Patras, 30200 Mesolongi, Greece; gbourantas@upatras.gr

**Keywords:** cerebral venous system, computational fluid dynamics, deep cerebral vein thrombosis, wall shear stress, patient-specific modeling, personalized medicine

## Abstract

**Background/Objectives**: Deep cerebral vein thrombosis (DCVT) is a rare cerebrovascular condition that can result in absence of major venous sinuses. This study uses patient-specific computational fluid dynamics (CFD) to quantify hemodynamic changes in acquired DCVT, focusing on venous outflow redistribution, pressure, and wall shear stress (WSS). **Methods**: Three-dimensional models of cerebral venous sinuses were reconstructed from magnetic resonance venography (MRV) for a DCVT patient and normal control. Steady-state CFD simulations used physiological inflows with laminar flow assumptions. Sensitivity analyses tested hyperemic conditions and blood rheology effects. **Results**: In normal anatomy, flow split 70% through superior sagittal sinus and 30% through straight sinus. In DCVT, all flow was rerouted through the superior sagittal sinus. Surprisingly, pressure drop was lower in DCVT (0.67 mmHg vs. 1.3 mmHg in normal). WSS increased moderately in the DCVT superior sagittal sinus (~2.5 Pa peak) but remained within physiological ranges. Under hyperemic conditions, pressures and WSS stayed below pathological thresholds. **Conclusions**: DCVT redirects venous outflow without pathological pressure or WSS elevations, demonstrating remarkable venous system resilience through collateral compensation. This patient-specific CFD framework enables individualized hemodynamic assessment, contributing to personalized medicine approaches for rare cerebrovascular conditions.

## 1. Introduction

The cerebral venous system drains deoxygenated blood from brain parenchyma through the following two main pathways: superficial cortical veins emptying into dural sinuses (primarily the superior sagittal sinus) and deep veins converging into the great vein of Galen and straight sinus [1,2,3]. These pathways normally converge at the torcular Herophili, distributing blood into transverse and sigmoid sinuses toward the internal jugular veins [4].

Deep cerebral vein thrombosis (DCVT) is a rare cerebrovascular condition accounting for 0.5% of strokes [5]. While traditionally attributed to congenital variations, emerging evidence suggests many cases of absent straight sinus result from acquired thrombotic conditions [6,7,8]. DCVT leads to absence of the straight sinus, internal cerebral veins, and vein of Galen, triggering hemodynamic changes and collateral development distinct from congenital variants [9].

Patient-specific computational fluid dynamics (CFD) provides unique insights into cerebral hemodynamics by reconstructing vascular anatomy from imaging and simulating blood flow [10,11,12]. CFD enables quantification of velocity fields, pressure distributions, and wall shear stresses difficult to measure in vivo, particularly valuable for rare conditions like DCVT where clinical data are limited [13].

This study applies CFD to quantify cerebral venous outflow changes when the straight sinus is absent due to thrombosis. We hypothesize that while the absence of the straight sinus will alter flow patterns, the venous network may compensate through alternative pathways to maintain drainage. Key questions include: How is flow redistributed when deep venous drainage is rerouted? What changes occur in pressure and wall shear stress distribution?

The patient-specific model represents a 35-year-old female presenting with headaches and visual disturbances. MRV confirmed DCVT with absence of straight sinus, internal cerebral veins, and vein of Galen, accompanied by a prominent falcine sinus as collateral pathway [7]. Conservative management with anticoagulation resulted in asymptomatic recovery at 6-month follow-up with normal intracranial pressure [5].

## 2. Materials and Methods

### 2.1. Imaging and Geometric Modeling

Patient-specific venous sinus geometry was obtained from high-resolution 3D phase-contrast MRV (0.9 × 0.9 × 1.6 mm^3^ resolution, ~500 slices). The patient showed absent straight sinus and deep veins consistent with DCVT. All imaging data were anonymized with appropriate institutional review.

Segmentation employed combined automatic and manual techniques using the Vascular Modeling Toolkit (VMTK) in 3D Slicer (v5.8.0, Slicer community, www.slicer.org; developed by the surgical planning laboratory Brigham and Women’s Hospital, Boston, MA, USA) [14]. Vessel enhancement filters highlighted tubular structures, followed by intensity thresholding to capture major venous channels. Manual segmentation added missing portions through slice-by-slice inspection.

**Figure 1:**
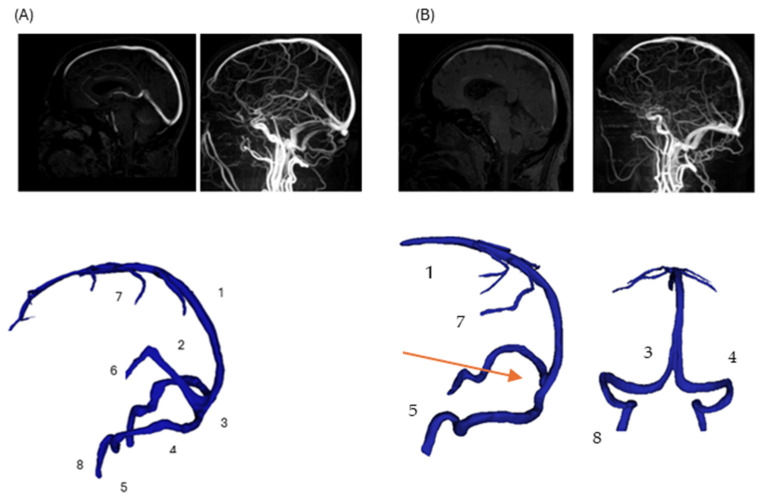
Comparison of normal and pathological cerebral venous anatomy. (Panel A): Three-dimensional reconstruction of normal cerebral venous anatomy, with major veins labeled (1—superior sagittal sinus; 2—straight sinus; 3—confluence of sinuses; 4—transverse sinus; 5—sigmoid sinus; 6—internal cerebral veins; 7—superior cerebral veins; 8—jugular vein). (Panel B): Corresponding 3D reconstruction of the patient-specific model with DCVT case. The straight sinus (2) and internal cerebral veins (6) are thrombosed and not visualized; their anatomical locations are indicated with arrow for reference. Other veins remain intact and are labeled consistently with (Panel A).

A normal venous anatomy model from an adult without abnormalities served as control, featuring intact internal cerebral veins, vein of Galen, and straight sinus. Surface models underwent smoothing to remove artifacts, with inlets defined at superior sagittal sinus (and straight sinus for normal model) and outlets at internal jugular veins. Comparison of the two reconstructed geometries of the two patients is shown in (Figure 1).

**Figure 2:**
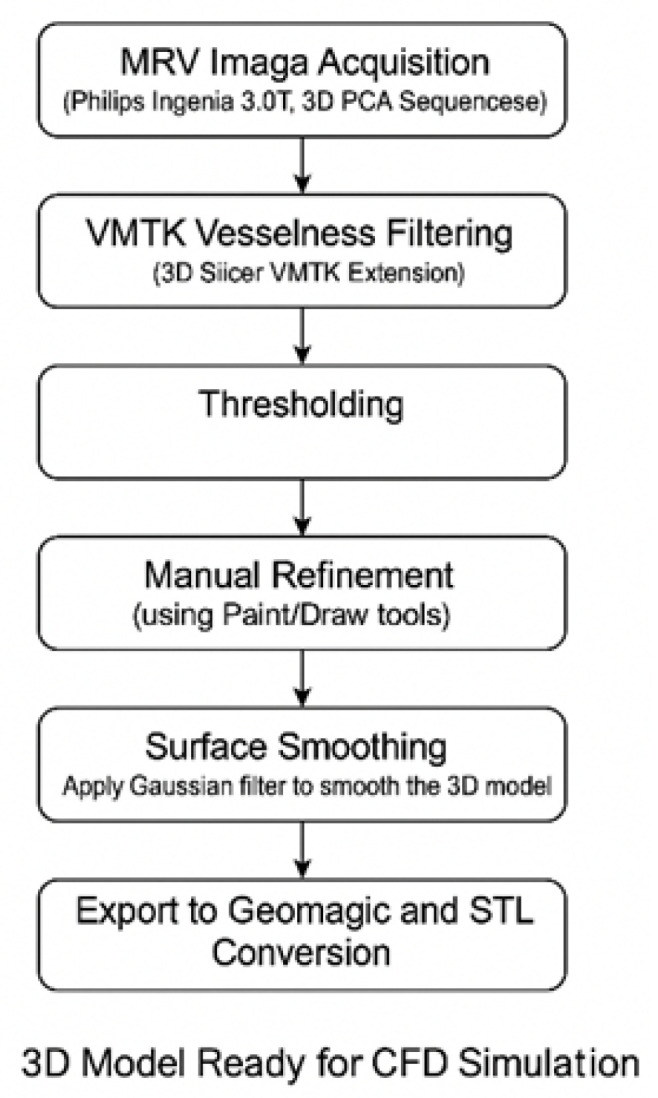
Visual summary of the image-based modeling workflow used for 3D reconstruction of cerebral venous anatomy. MRV data were processed using a hybrid segmentation pipeline: VMTK and intensity thresholding were followed by manual refinement to ensure anatomical completeness. Surface smoothing and mesh preparation were then applied to produce CFD-compatible geometries for both normal and DCVT cases.

This illustration reflects the structural disruption caused by DCVT, consistent with typical MRI findings in venous sinus thrombosis (e.g., absence of flow void on MRI due to thrombosis) [15]. Notably, venous collaterals are preserved, highlighting post-thrombotic remodeling and collateral adaptation seen in acute DCVT cases [10]. The overall pipeline to prepare the final geometries is presented in Figure 2.

### 2.2. Computational Setup

Unstructured tetrahedral meshes captured complex sinus geometries with refined resolution in narrow channels and high-curvature regions. Mesh independence studies confirmed convergence at ~790,000 elements, providing optimal accuracy–efficiency balance. Final meshes contained 600–800 k cells with high quality (minimum angles > 20°, aspect ratios < 5) [12]. CFD simulations used ANSYS Fluent, v 2025, Canonsburg, PA, USA with steady-state Navier–Stokes equations under laminar flow assumptions, validated by comparison with turbulence models. Convergence criteria required residuals < 1 × 10^−4^ and mass imbalances < 0.1% [11,12].

### 2.3. Boundary Conditions and Blood Flow Parameters

Inlet Conditions: Based on phase-contrast MRI studies, physiological flow rates were 4.16 mL/s at superior sagittal sinus and 1.5 mL/s at straight sinus for normal anatomy (70:30 split, total 5.66 mL/s) [16]. For DCVT, all 5.66 mL/s entered through superior sagittal sinus only, simulating deep venous rerouting via collaterals [17].

In additional simulations, we explored a more detailed inlet condition by distributing flow among multiple smaller tributary veins in the normal anatomy (e.g., adding separate inflows for cortical veins, internal cerebral veins, etc.). This “full-network” inlet configuration was used to verify that lumping the inflow into the main sinuses (as performed in the primary simulations) did not significantly alter the results. The total flow was kept the same, only split into more entry points according to the literature values. The simplified two-inlet approach was found to be sufficient for capturing the major hemodynamics, as validated by comparing it with the full-network model.

Outlet Conditions: Constant pressure (5 mmHg) at both internal jugular veins represented normal venous pressure, allowing natural flow distribution based on anatomical resistance [8].The outlet pressure of 5 mmHg was selected based on physiological jugular venous pressure values (3–7 mmHg) reported in the literature for supine subjects [1,8,16]. Although a formal sensitivity sweep was not performed, previous modeling studies and preliminary tests indicate that moderate variations within this range have minimal influence on relative flow distribution and pressure gradients. Therefore, symmetric outlet pressures were used to isolate the geometric effects of deep cerebral vein thrombosis (DCVT) while maintaining physiologically realistic boundary conditions.

Blood Properties: Newtonian fluid with density 1060 kg/m^3^ and viscosity 3.5 cP, appropriate for low-shear venous flows. Sensitivity analyses tested non-Newtonian rheology using Carreau–Yasuda and Cross models. Vessel walls were treated as rigid no-slip boundaries [18], a common assumption in steady-state venous CFD that isolates geometric effects while recognizing limitations discussed in Section 4.4.

Sensitivity Analysis: Flow was increased by ~39% to simulate hyperemic conditions and assess system capacity under increased cerebral blood flow [19].

### 2.4. Numerical Solution and Solver Setup

The CFD simulations were carried out in ANSYS Fluent (ANSYS Inc., Canonsburg, PA, USA). Steady-state flow was solved with second-order discretization schemes. Convergence criteria required residuals < 1 × 10^−4^ and mass imbalances < 0.1%.

Since venous pulsatility is damped compared to arterial flow, the steady-state assumption is commonly adopted in cerebral venous CFD [10]. Additionally, a turbulence model (k–ω SST) was tested for baseline conditions but yielded essentially identical results, confirming that flow remained laminar (Reynolds numbers in the SSS on the order of a few hundred) [18].

## 3. Results

### 3.1. Baseline Flow Distribution

**Figure 3:**
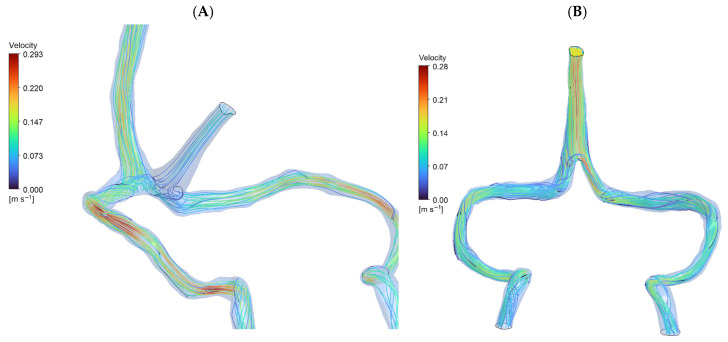
CFD streamlines (**A**): Normal Cerebral Anatomy showing venous blood flow entering from the superior sagittal sinus and straight sinus (approximately 70:30 distribution), converging at the torcular Herophili to form a stable recirculation zone. Outflow proceeds predominantly to the right transverse sinus (60–65%). Peak velocity: 0.293 m/s. (**B**): Deep Cerebral Vein Thrombosis (DCVT) showing flow entering exclusively via the superior sagittal sinus (no straight sinus inflow). This yields unidirectional flow without recirculation. Flow is split with a slight leftward bias (~55% left vs. ~45% right). Peak velocity: 0.281 m/s.

Normal anatomy exhibited expected flow division with superior sagittal sinus (SSS) and straight sinus (STS) streams meeting at torcular Herophili. The flow split approximately 70:30 between superficial and deep routes, with right-dominant outflow (60–65% right vs. 35–40% left transverse sinus), consistent with clinical observations [6]. In DCVT anatomy, entire venous return routed through superior sagittal sinus alone. Without straight sinus contribution, flow pattern simplified to unidirectional streamlines from SSS bifurcating toward transverse sinuses (Figure 3). This produced more symmetrical flow distribution, actually favoring left side (55% left vs. 45% right), opposite to normal dominance pattern. Table 1 presents a comparison between the two anatomies.

Peak velocity in DCVT was higher in SSS region (0.28 m/s vs. 0.11 m/s normal) due to entire output channeling through single sinus initially, then decreasing as flow divided downstream.

### 3.2. Pressure and Wall Stress Distribution

**Figure 4:**
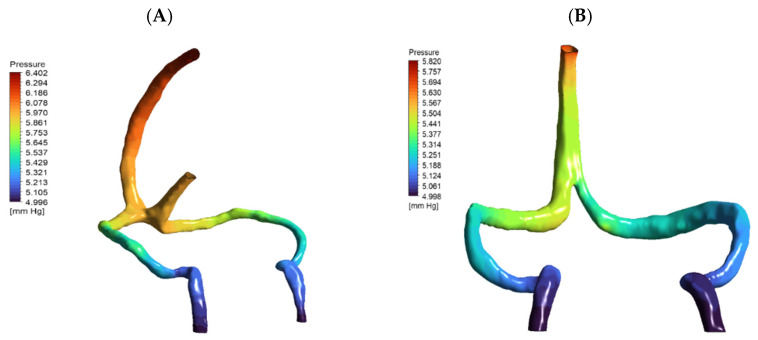
Pressure distribution maps (mmHg) for (**A**) normal and (**B**) DCVT venous anatomies under baseline flow (5.4 mL/s), rendered from the same viewpoint. Both panels are color-coded by pressure. In (**A**) (normal anatomy), flow enters via both the superior sagittal and straight sinuses, yielding a total pressure drop (ΔP) of 1.319 mmHg. In (**B**) (DCVT anatomy), flow enters only via the superior sagittal sinus; this configuration produces a more streamlined single-stream flow with reduced viscous losses and near-symmetric outlet pressures, and a lower ΔP of 0.674 mmHg.

Surprisingly, DCVT anatomy showed lower overall pressure drop (0.67 mmHg vs. 1.3 mmHg normal) (Figure 4). This counter-intuitive finding resulted from more streamlined single-stream flow eliminating energy losses from stream mixing at confluence. The absence of colliding flows reduced turbulence and viscous losses, making the singular pathway more hydraulically efficient.

**Figure 5:**
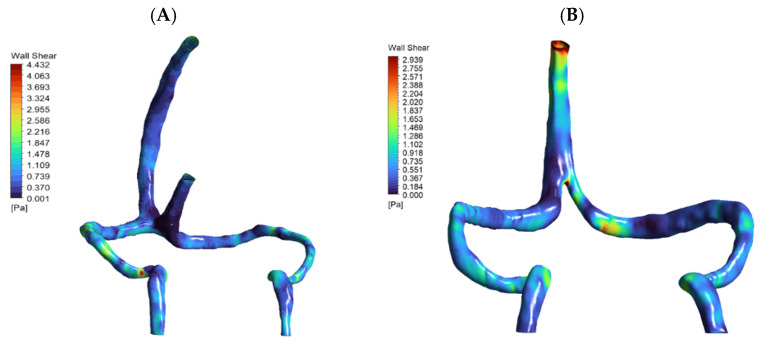
Contour maps of wall shear stress (WSS) on the inner walls of patient-specific cerebral venous sinuses for (**A**) the normal anatomy and (**B**) the DCVT case with an absent straight sinus. The color bar shows local WSS in Pascals (Pa). In the normal model, the highest shear (~4.4 Pa) occurs in the sigmoid sinus (at the curvature/narrowing). In the DCVT model (no straight sinus), all flow is rerouted through the superior sagittal sinus (SSS), so the WSS peak (~2.9 Pa) shifts to the SSS–transverse sinus bifurcation. In both cases the WSS values remain low (only a few Pascals), consistent with physiological venous shear stress levels. Thus, these maps illustrate how loss of the straight sinus modestly raises shear in the SSS and redistributes flow, without producing abnormally high WSS.

Wall shear stress remained within physiological limits in both anatomies. Normal model showed peak WSS ~3.9 Pa in sigmoid regions, while DCVT peak was ~2.5 Pa at SSS-transverse sinus bifurcation (Figure 5). Importantly, none of the observed WSS values approached pathological thresholds (>15–20 Pa) reported to cause endothelial activation [20].

### 3.3. Effect of Increased Flow (Hyperemia)

Under a 39% flow increase, normal anatomy pressure drop rose from 1.3 to 1.8 mmHg, while DCVT rose from 0.67 to 1.05 mmHg (Table 2). Although the DCVT model showed slightly greater sensitivity, absolute values remained well below intracranial hypertension thresholds.

**Figure 6:**
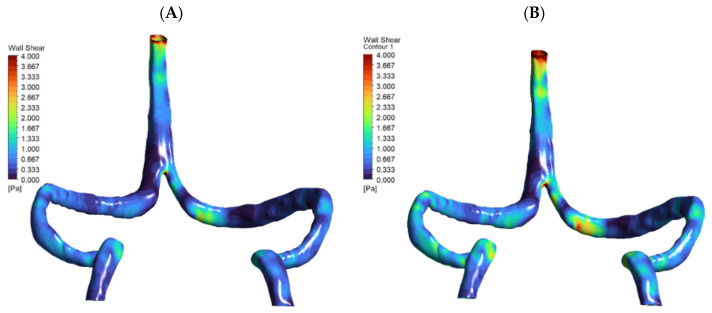
Wall shear stress (WSS) distribution at baseline and +30% flow. Two-panel WSS maps for the DCVT model are shown: (**A**) baseline flow and (**B**) after a 30% flow increase, using a 0–4 Pa color scale. The higher-flow case produces noticeably elevated WSS in the superior sagittal sinus and at the torcular (confluence) junction, reflecting the hyperemic loading.

Peak WSS under hyperemia reached ~12 Pa in DCVT (Figure 6), which is elevated but still below the 15 Pa threshold known to trigger endothelial dysfunction [20]. This indicates that the venous system can tolerate flow increases without entering pathological ranges.

### 3.4. Comprehensive Cerebral Venous Network Analysis

To see how much smaller tributaries affect our results, we ran an extended simulation of the normal anatomy that included the superficial middle cerebral veins, internal cerebral veins, and basal veins of Rosenthal, with flow distributed realistically among these vessels [10,12].

**Figure 7:**
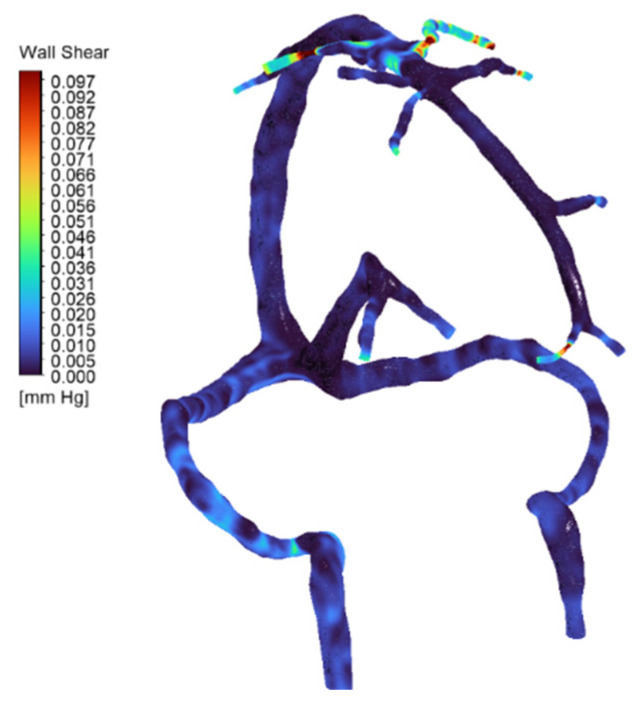
Wall shear stress (WSS) distribution in the full cerebral venous network for the normal anatomy (full-network CFD simulation). The map (color-coded with blue for low values) shows generally low venous shear stress throughout most of the network. Localized high-shear regions appear at the junctions of the superficial middle cerebral veins (SMCVs), where inflow is greatest and vessel caliber is reduced—values reach on the order of ~1.3 Pa at these SMCV junctions. These peaks (indicated by warmer colors) correspond to the elevated flow and possible geometric narrowing at those tributaries.

**Figure 8:**
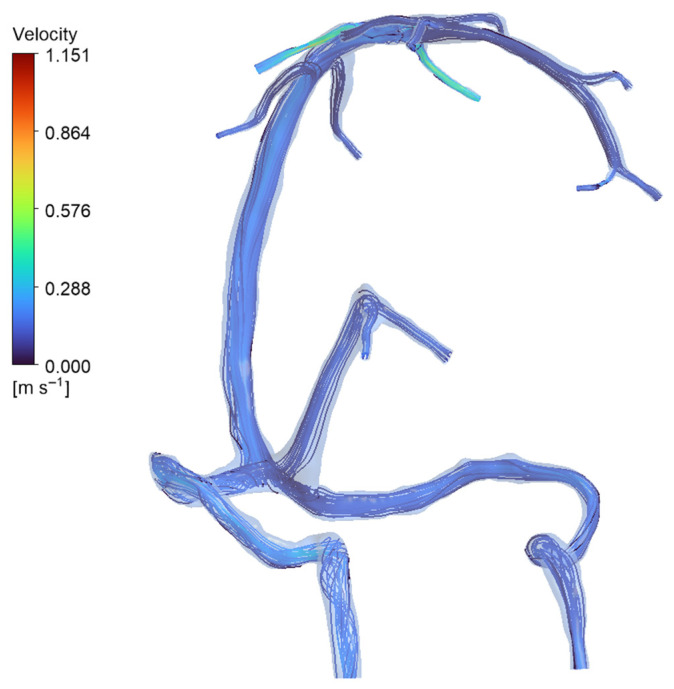
Streamline visualization of venous inflow in the normal anatomy (full-network simulation). Streamlines (plotted from the inlet tributaries) show how blood enters via multiple veins and is carried into the dural sinuses. In the normal model, inflow is distributed among the major tributaries—specifically the superior cerebral veins (SCVs), internal cerebral veins (ICVs), and basal veins of Rosenthal (BVRs)—rather than coming from a single source. The map confirms the parallel contributions of these SCVs, ICVs, and BVRs to overall venous drainage in the healthy anatomy, with each major vein supplying flow into the sinus network.

Adding these smaller tributaries did increase wall shear stress locally by about 27%, with peaks around 1.3 Pa at the junctions where these veins meet (see Figure 7 and Table 3). Interestingly though, the big-picture pressure patterns stayed quite stable, varying by less than 0.3 mmHg. This makes sense given how the venous network naturally equalizes pressure. What this tells us is that while including detailed tributary anatomy matters for understanding local hemodynamic hotspots, our simplified two-inlet approach used in the main simulations does a good job of capturing the overall flow behavior and pressure distribution in the major sinuses [6]. Velocity streamlines are presented in Figure 8.

### 3.5. Rheological Effects

**Figure 9:**
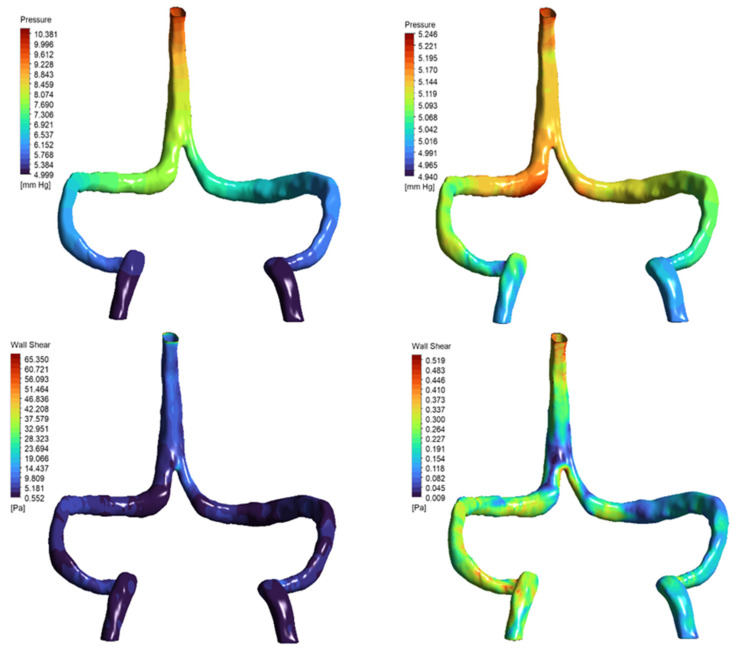
Pressure and wall shear stress distributions under non-Newtonian rheology models. Top row: Static pressure distribution in the DCVT model using Carreau–Yasuda (left) and Cross (right) viscosity models. The Carreau–Yasuda simulation shows a steeper pressure gradient (ΔP ≈ 4.7 mmHg) compared to the more uniform gradient observed with the Cross model (ΔP ≈ 0.2 mmHg). Bottom row: Wall shear stress (WSS) fields reveal localized high shear concentrations in the Carreau–Yasuda case (peak WSS ≈ 65 Pa), in contrast to the smoother, physiologically consistent distribution seen with the Cross model (peak WSS < 0.5 Pa). These results illustrate the impact of rheological assumptions on local hemodynamic metrics in venous CFD simulations.

Newtonian and mild shear-thinning (Cross) models yielded physiologically realistic results differing by only a few percent. However, aggressive shear-thinning (Carreau–Yasuda) produced unrealistic values (pressure drops > 4 mmHg, WSS > 60 Pa), highlighting sensitivity to rheological assumptions in venous CFD [18]. The results are presented in Table 4 and Figure 9.

## 4. Discussion

### 4.1. Hemodynamic Adaptation Mechanism

Our patient-specific CFD simulations highlight the remarkable adaptability of the cerebral venous system in deep cerebral vein thrombosis (DCVT). Complete rerouting of deep venous return through collateral pathways into the superior sagittal sinus occurred without evidence of system overload. The lower pressure drop in DCVT compared to normal anatomy suggests that a singular collateralized pathway can be as hydraulically efficient as dual venous routes when adequate compensatory channels are present [21,22]. The fluid-mechanical basis for this efficiency involves three factors. First, eliminating flow convergence removes the dissipative mixing zone that forms when opposing streams meet at the confluence (analogous to T-junction losses in pipe networks). Second, the pressure drop (ΔP) in the DCVT model scales only with SSS resistance, while the normal model experiences additive losses from both inflow paths plus mixing. Third, unidirectional flow maintains laminar profiles (Reynolds number ~200 in SSS), whereas confluence turbulence (even mild) increases viscous dissipation. These mechanisms collectively explain why complete occlusion with collateral bypass can paradoxically reduce overall venous resistance compared to the dual-pathway normal anatomy.

This adaptation underscores a critical physiological principle: collateral remodeling is not merely an anatomical curiosity, but an effective safeguard for maintaining venous outflow capacity [6,23]. The patient’s favorable clinical recovery, despite severe anatomical disruption, aligns with our CFD prediction of preserved hemodynamic stability.

### 4.2. Clinical Significance

The absence of pathological pressure elevations or excessive wall shear stress (WSS) in the DCVT model provides mechanistic insight into why many patients with adequate collaterals recover without neurological decline. Even under simulated hyperemic conditions, venous pressures and WSS remained below thresholds known to trigger endothelial activation or intracranial hypertension [20]. These findings suggest that hemodynamic resilience, rather than thrombosis alone, may determine outcomes in DCVT.

Such observations support the role of patient-specific CFD as a potential prognostic tool. Quantitative metrics such as predicted pressure drop, collateral flow distribution, and local WSS may improve risk stratification in patients with venous sinus thrombosis [13,24]. While conservative anticoagulation remains first-line therapy for most DCVT cases, recent meta-analyses indicate that endovascular thrombectomy may benefit severe presentations with worsening symptoms despite medical management [25]. The present CFD framework could potentially inform patient selection by identifying cases with hemodynamic reserve capacity that may respond to conservative treatment versus those requiring aggressive intervention.

### 4.3. Comparison to Prior Studies

Previous CFD investigations of cerebral venous flow have largely focused on transverse or sigmoid sinus stenosis, where partial narrowing elevates venous pressures [24,26]. In contrast, our study addresses the underexplored scenario of complete straight sinus occlusion, demonstrating that when effective collateral bypass routes exist, global hemodynamics can remain within safe limits. This finding bridges an important knowledge gap between stenosis-driven impedance and collateral-mediated compensation.

### 4.4. Limitations

This study has several limitations. While this N = 1 study limits population-level conclusions, it establishes proof of concept for patient-specific venous CFD in rare pathologies. The rigorous sensitivity analyses (mesh independence, rheology testing, hyperemia simulation) ensure the methodology is reproducible when applied to larger cohorts. Venous walls and bridging veins were modeled as rigid. In reality, bridging veins behave as Starling resistors with partial collapsibility and compliance on the order of 2–3% area change per mmHg, which buffers intracranial pressure fluctuations [27]. Neglecting this mechanism may overestimate local pressure and wall shear stress. Future fluid–structure interaction (FSI) extensions could explicitly model this compliant behavior to enhance physiological realism. The present model omits vertebral and emissary venous routes, which in vivo may contribute up to 30% of total cranial venous drainage in the supine position [1]. Their omission could slightly overestimate jugular velocities and wall shear stress, yet overall pressure gradients (<2 mmHg) remain within physiological ranges. Incorporating these pathways in future CFD frameworks will improve the completeness of cerebral venous modeling.

Third, the exclusion of smaller bridging and cortical collaterals represents a limitation worth addressing. We tested the impact of this simplification in the normal anatomy (see Section 3.4), where adding smaller tributaries increased local wall shear stress by approximately 27% at junctions while overall pressure patterns remained stable. However, we did not perform analogous testing on the DCVT model due to imaging resolution constraints that prevented reliable segmentation of collateral pathways in the patient. Consequently, our DCVT simulations may underestimate true drainage capacity and localized hemodynamic phenomena at collateral junctions. Nevertheless, the model captures the major venous drainage pathways responsible for bulk flow dynamics, and the patient’s favorable clinical outcome suggests these pathways provided adequate drainage. Future studies with higher-resolution imaging or idealized collateral geometries would better quantify collateral contributions in DCVT hemodynamics. Fourth, the assumption of steady-state flow neglects venous pulsatility, which may influence local hemodynamic fluctuations. Assuming normal jugular function and fixed outlet pressures may overlook the effects of asymmetric or impaired outflow, which should be explored in future work.

### 4.5. Future Directions

Future work should incorporate multiple patient-specific geometries to establish statistical robustness. Pulsatile simulations combined with 4D phase-contrast MRI validation would allow closer alignment with in vivo physiology. In addition, fluid–structure interaction (FSI) modeling could capture the role of bridging vein compliance as Starling resistors, which buffer sudden changes in pressure and flow. Incorporating FSI modeling and coupling 3D/with lumped-parameter Starling-resistor elements [28] could further capture venous wall compliance and dynamic pressure buffering. Such enhancements would strengthen the translational potential of CFD in cerebrovascular medicine.

## 5. Conclusions

This patient-specific CFD analysis demonstrates that cerebral venous drainage remains highly efficient despite severe DCVT, owing to collateral compensation. Compared to normal anatomy, the DCVT model showed a halved pressure drop and WSS values within physiological limits, reflecting the resilience of the venous network.

These findings provide a mechanistic explanation for the favorable outcomes observed in patients with well-developed collaterals and highlight the value of CFD in assessing hemodynamic reserve. Beyond descriptive imaging, CFD enables individualized prediction of pressure and shear stress responses under variable flow conditions, which could guide prognosis and therapy planning.

By establishing that complete venous sinus occlusion with collateral pathways does not necessarily lead to pathological hemodynamics, our study reframes the clinical interpretation of DCVT from one of inevitable risk to one of conditional resilience. Clinically, these findings suggest that anatomical imaging alone, showing absent straight sinus, is insufficient for risk stratification. CFD-derived metrics (pressure drop, WSS distribution, hyperemic reserve) may identify which patients can be safely managed conservatively versus those requiring intervention. As computational tools become clinically accessible, integration of patient-specific hemodynamic assessment into diagnostic workflows may enable truly personalized treatment decisions in rare cerebrovascular pathologies. Integration of CFD metrics into prognostic models may enable precision medicine approaches, allowing clinicians to tailor treatment based not only on anatomy but also on predicted hemodynamic consequences.

From a broader perspective, this work demonstrates how computational modeling can bridge the gap between rare clinical observations and mechanistic understanding, offering a pathway toward personalized cerebrovascular care.

## Figures and Tables

**Table 1:** Key baseline hemodynamic metrics in the normal vs. DCVT anatomy. (Values in parentheses are approximate from simulation).

Metric	Normal Anatomy	DCVT Anatomy
SSS vs. STS inflow split	~70% SSS/30% STS	100% SSS/0% STS
Jugular outflow split (right:left)	~1.6:1 (right dominant)	~0.8:1 (left dominant)
Peak velocity in sinuses	0.11 m/s (SSS region)	0.28 m/s (SSS region)
Pressure drop (SSS inlet to IJVs)	~1.3 mmHg	~0.67 mmHg
Average SSS wall shear stress (WSS)	~1.5 Pa	~1.8 Pa
Peak WSS	~3.9 Pa (near sigmoid)	~2.5 Pa (near SSS outlet)

**Table 2:** DCVT model pressure drop vs. flow.

Flow Rate (mL/s)	Pressure Drop (mmHg)	Flow Condition
5.4	0.674	Baseline
5.94	0.766	+10% Flow
6.21	0.81	+15% Flow
6.48	0.862	+20% Flow
6.75	0.911	+25% Flow
7.02	0.96	+30% Flow
7.25	1.00	+34% Flow
7.5	1.051	+39% Flow

**Table 3:** Pressure distribution across major cerebral venous tributaries in the normal full-network model. Values represent average static pressures at the outlet of each tributary under baseline inflow. The data highlight minor inter-tributary pressure differences, with generally uniform values across the network, reflecting stable drainage conditions. This confirms efficient pressure equalization in the healthy cerebral venous system under physiologic flow.

Vessel	Pressure (Pa)	Pressure (mmHg)
Internal Cerebral Vein (Left)	837.71	6.28
Internal Cerebral Vein (Right)	846.98	6.35
Superficial Middle Cerebral Vein (Left)	1452.61	10.90
Superficial Middle Cerebral Vein (Right)	1444.07	10.84
Superior Cerebral Vein (Midline)	951.33	7.14
Superior Cerebral Vein (Left)	952.90	7.15
Superior Cerebral Vein (Left)	1074.52	8.06
Superior Cerebral Vein (Left)	882.33	6.62
Superior Cerebral Vein (Right)	1277.22	9.60
Superior Cerebral Vein (Right)	991.76	7.44
Superior Cerebral Vein (Right)	934.26	7.01
Superior Cerebral Vein (Right)	907.20	6.81

**Table 4:** Summary of hemodynamic parameters under Newtonian, Carreau–Yasuda, and Cross models (DCVT).

Quantity	Newtonian	Carreau-Yasuda	Cross	% Diff (vs. CY)	% Diff (vs. Cross)
Peak Velocity (SSS, m/s)	0.1658	0.1658	0.1658	0%	0%
Peak Velocity (fluid, m/s)	0.2830	0.349	0.225	+23.3%	−20.5%
Area-Averaged WSS (Pa)	1.80	4.04	0.204	+124.4%	−88.7%
Pressure Drop (mmHg)	0.679	4.725	0.2	+595.6%	−70.5%

## Data Availability

The original contributions presented in this study are included in the article. Further inquiries can be directed to the corresponding author.

## Abbreviations

The following abbreviations are used in this manuscript:

ICVs	Internal Cerebral Veins
DCVT	Deep Cerebral Vein Thrombosis
ICP	Intracranial Pressure
IIH	Idiopathic Intracranial Hypertension
CSF	Cerebrospinal Fluid
IJVs	Internal Jugular Veins
MRV	Magnetic Resonance Venography
SCVs	Superior Cerebral Veins
SSS	Superior Sagittal Sinus
STS	Straight Sinus
WSS	Wall Shear Stress
CFD	Computational Fluid Dynamics
